# Traditional Chinese Medicine Shuang Shen Ning Xin Attenuates Myocardial Ischemia/Reperfusion Injury by Preserving of Mitochondrial Function

**DOI:** 10.1155/2014/180965

**Published:** 2014-06-16

**Authors:** Xueli Li, Jianxun Liu, Li Lin, Yujie Guo, Chengren Lin, Cuixiang Zhang, Bin Yang

**Affiliations:** ^1^Beijing University of Chinese Medicine, Beijing 100029, China; ^2^Institute of Basic Medical Sciences, Xiyuan Hospital, China Academy of Chinese Medical Sciences, No. 1 Xiyuan Caochang, Haidian District, Beijing 100091, China

## Abstract

To investigate the potential cardioprotective effects of Shuang Shen Ning Xin on myocardial ischemia/reperfusion injury. Wistar rats were treated with trimetazidine (10 mg/kg/day, ig), Shuang Shen Ning Xin (22.5, 45 mg/kg/day, ig), or saline for 5 consecutive days. Myocardial ischemia/reperfusion injury was induced by ligation of the left anterior descending coronary artery for 40 min and reperfusion for 120 min on the last day of administration. It is found that Shuang Shen Ning Xin pretreatment markedly decreased infarct size and serum LDH levels, and this observed protection was associated with reduced myocardial oxidative stress and cardiomyocyte apoptosis after myocardial ischemia/reperfusion injury. In addition, further studies on mitochondrial function showed that rats treated with Shuang Shen Ning Xin displayed decreased mitochondrial swelling and cytosolic cytochrome c levels, which were accompanied by a preservation of complex I activities and inhibition of mitochondrial permeability transition. In conclusion, the mitochondrial protective effect of Shuang Shen Ning Xin could be a new mechanism, by which Shuang Shen Ning Xin attenuates myocardial ischemia/reperfusion injury.

## 1. Introduction

Myocardial ischemia/reperfusion (MI/R) has been shown to result in mitochondrial dysfunction, and the importance of mitochondria as both targets and mediators of MI/R injury is well recognized [1, 2]. The mitochondrion is a vital component in cellular energy metabolism and intracellular signaling cascades. As the high energy requirement of contracting cardiac myocytes, the mitochondria cover 30% of the cell's total volume providing energy via oxidative phosphorylation, and as a result these cells are particularly vulnerable to mitochondrial defects [3]. During myocardial ischaemia and reperfusion, mitochondria can quickly turn into death-promoting organelles by depressing respiratory chain complex activity, disrupting adenosine triphosphate synthesis, releasing pro-death proteins, producing reactive oxygen species, and inducing the mitochondrial permeability transition pore (mPTP) opening [4]. Thus, mitochondrial dysfunction is widely acknowledged as an important event in necrotic and apoptotic cell death and is emerging as a key mediator of MI/R injury. In this context, the preservation of mitochondrial integrity and function is a potential therapeutic strategy to limit MI/R injury and attenuate the disease process.

Traditional Chinese medicine (TCM) has unique theories of etiology, diagnosis, and treatment. According to TCM, the pathogenesis of MI/R injury is usually diagnosed as Qi stagnation and blood stasis [5, 6]. Shuang Shen Ning Xin (SSNX), a traditional Chinese medicine for invigorating Qi, activating blood circulation, and relieving pain, has been developed for the treatment of coronary heart diseases. The SSNX is composed of three kinds of effective fractions in certain proportion including total ginsenosides, total salvianolic acids, and total alkaloids which are isolated from* Ginseng* Radix et Rhizoma,* Salviae miltiorrhizae* Radix et Rhizoma, and* Corydalis* Rhizoma, respectively, and the quality control of SSNX is satisfactory. Experimental studies have shown that SSNX can effectively inhibit myocardial infarction and preserve left ventricular structure and function in a porcine myocardial ischemia model [7–9]. Moreover, these beneficial effects can be partly attributed to improving cardiac energy metabolism during ischemia or reperfusion [10]. These results indicate that mitochondria may be a potential therapeutic target of SSNX to reduce cell damage during MI/R. Therefore, the aim of this study is to ascertain whether the mitochondrial protection was involved in the above cardioprotective effects of SSNX.

## 2. Materials and Methods

### 2.1. Drugs and Reagents

SSNX was provided by Institute of Basic Medical Sciences of Xiyuan Hospital (Beijing, China). In brief, three kinds of Chinese herbs including* Ginseng* Radix et Rhizoma,* Salviae miltiorrhizae* Radix et Rhizoma, and* Corydalis* Rhizoma were extracted by alcohol according to a standard method to obtain “full components” and were separated by macroporous resin column to isolate target effective fractions such as total ginsenosides (containing in % the following: 18.6 ginsenoside Rg_1_; 6.2 ginsenoside Re; 14.6 ginsenoside Rb_1_; 2.6 ginsenoside Rd; 7.7 ginsenoside Rc; 2.5 ginsenoside Rb_2/3_; 3.7 ginsenoside Rf), total salvianolic acids (containing in % the following: 1.3 salvianolic acid A; 55.1 salvianolic acid B; 3.0 rosmarinic acid; 2.9 lithospermic acid), and total alkaloids (containing in % the following: 0.8 berberine; 3.5 tetrahydropalmatine; 5.0 palmatine; 17.4 dehydrocorydaline; 2.6 corydaline; 0.4 worenine; 3.1 protopine; 1.2 epiberberine; 2.0 tetrahydrocolumbamine; 3.4 coptisine; 2.2 A-allocryptopine; 3.6 glaucine; 3.0 Jatrorrhizine; 3.1 tetrahydrojatrorrhizine; 0.2 canadine), respectively. SSNX was composed of these three kinds of effective fractions in certain proportion.

Trimetazidine (TMZ) was produced by Servier Pharmaceutical Co., Ltd., Tianjin, China. Nitroblue tetrazolium (NBT) was purchased from Amresco (Solon, OH, USA). The in situ cell apoptosis detection kit was purchased from Boster Biological Technology (Wuhan, China). The assay kit of lactate dehydrogenase (LDH) was purchased from Hitachi High-Tech (Japan). The assay kits of malondialdehyde (MDA) and glutathione peroxidase (GSH-Px) were purchased from Nanjing Jiancheng Bioengineering Institute (Nanjing, China). The assay kits of Cytochrome c (Cyt c) Protein Quantity and Complex I Enzyme Activity Microplate were purchased from Abcam (Cambridge, UK). The rabbit anticleaved caspase-3 was purchased from Cell Signaling Technology (Beverly, MA, USA).

### 2.2. Animal Treatment

Wistar rats (SPF grade, male, 230–280 g) were purchased from the Vital River Laboratories (VRL, Beijing, China). The animals were kept in rooms maintained at 23 ± 2°C in a 12 h light/dark cycle and were fed a rodent standard diet with free access to water following international recommendations. All animal experiments in this study were performed in accordance with China Academy of Chinese Medical Sciences Guide for Laboratory Animals that conforms to the Guide for the Care and Use of Laboratory Animals published by the U.S. National Institutes of Health (NIH Publications number 85-23, revised in 1996). Rats were randomly divided into five groups and treated as follows: (1) the sham group; (2) the I/R group; (3) the I/R group treated with TMZ solution at doses of 10 mg/kg TMZ; (4) the I/R group treated with SSNX solution at doses of 22.5, 45 mg/kg SSNX, respectively. TMZ or SSNX was given intragastrically one time a day for five consecutive days, while the sham and I/R groups were given normal saline.

One hour after the last treatment period, rats were anesthetized with chloral hydrate (350 mg/kg, ip) before operation. The surgical procedure for MI/R was performed as previously described [10]. Briefly, a parasternal incision was made by cutting the intercostal muscles between the left third and fourth ribs and the pericardium was excised to expose the heart. Then, myocardial ischemia was induced by ligation of the left anterior descending coronary artery at a point 1~2 mm inferior to the left auricle with a 6-0 silk suture. After 40 min of left coronary artery (LCA) ischemia the ligature was released to allow reperfusion for 2 h.

At the end of the reperfusion period, blood samples were drawn from the abdominal aorta and serum was separated by centrifugation at 1620 g for 10 min (Thermo Scientific IEC CL31R Multispeed Centrifuge) and analyzed for LDH activity. The hearts were subsequently excised and processed for biochemical, morphological, and molecular studies.

### 2.3. Assessment of Myocardial Infarct Size

At the end of the experiments, rat hearts were removed and irrigated with normal saline to wash out blood. The left ventricle was separated and sliced into 2 mm thick transverse sections which were incubated with 0.05% NBT solution (dissolved in phosphate buffer pH 7.2) at 37°C for 15 min to distinguish the viable myocardium from the necrotic myocardium. The viable tissue was stained dark blue by NBT, while the infarct portion without NBT staining remained red. Both sides of each section were photographed. The area measurement was performed by a computerized high-resolution pathological image analysis system (HPIAS-1000, Wuhan, China). Infarct size was calculated as a percentage of the left ventricle as follows: infarct size = total area of red tissue/area of the left ventricle × 100%.

### 2.4. Biochemical Studies

The 10% heart homogenate was prepared in ice-cold phosphate buffer (0.1 mol/L, pH 7.4) and centrifuged at 1620 g for 15 min at 4°C. The supernatant was used for determination of MDA levels and GSH-Px activities after the measurement of total protein content. Furthermore, the serum LDH levels were assayed spectrophotometrically with a commercial kit following the manufacturer's instructions.

### 2.5. Histological Analysis

Left ventricular heart tissues fixed in 10% phosphate-buffered formalin were embedded in paraffin, and serial sections (4 *μ*m thick) were cut using microtome (Leica RM 2125, Germany). Several sections were stained with hematoxylin and eosin (HE) and examined under a light microscope (Olympus BX51, Tokyo, Japan) for any histopathological changes. Other serial sections were used to determine myocardial apoptosis.

### 2.6. Determination of Myocardial Apoptosis by TdT-Mediated dUTP Nick End Labeling (TUNEL)

A TUNEL assay was performed with the commercially available in situ cell apoptosis detection kit, according to the manufacturer's instructions. Proteinase K (100 *μ*L) was added to each heart tissue slide for 15 min, and then slides were incubated with TdT and DIG-dUTP mixture labeling buffer at 37°C for 2 h. After the reactions were terminated, the slides were washed and incubated with hematoxylin to determine the number of nuclei. The TUNEL signals were observed with the Olympus BX51 microscope. In each slide, the TUNEL signals (brown signals) were mainly distributed around the border zone of the ischemic area in which five fields were randomly selected and the cells positive with TUNEL staining and hematoxylin staining (blue signals) were counted. Quantitative assessment of apoptosis was determined as a ratio of the number of TUNEL-positive cells to total nuclear number in each field.

### 2.7. Western Blot Analysis

Myocardium tissue samples were homogenized with ice-cold lysis buffer (Applygen Technologies Inc., Beijing, China). The lysates were centrifuged, and protein concentration was determined with BCA protein assay kit (Applygen Technologies Inc., Beijing, China). Protein extracts (10 *μ*g) were separated by electrophoresis on 12% sodium dodecyl sulfate polyacrylamide gel (SDS-PAGE) and then transferred onto polyvinylidene difluoride (PVDF) membranes. Blots were first blocked in 5% dry milk for 30 min and then incubated overnight at 4°C with primary antibody (rabbit anticleaved caspase-3 at 1 : 1000 dilution). The membranes were subsequently washed five times in 1 × Tris-buffer saline Tween 20 (TBST) buffer and then incubated with horseradish peroxidase- (HRP-) conjugated goat anti-rabbit secondary antibodies (dilution 1 : 40000; Cell Signaling). The protein bands were visualized with an enhanced chemiluminescent substrate (Applygen Technologies Inc., Beijing, China). Glyceraldehyde-3-phosphate dehydrogenase (GADPH) was chosen as a loading control to further assure the same volume for all the samples.

### 2.8. Identification of Cardiac Mitochondria with Transmission Electron Microscope

Electron microscopy was used to identify isolated mitochondria from the hearts. Isolated cardiac mitochondria were fixed in 2.5% glutaraldehyde in phosphate buffer (0.1 mol/L, pH 7.4) at 4°C. After rinsing in cacodylate buffer, mitochondrial pellets were postfixed in 1% cacodylate-buffered osmium tetroxide for 2 h at room temperature and then dehydrated in a graded series of ethanol solutions. The specimens were then embedded in Epon Araldite to block tissue and ultrathin sections. The sections were stained with uranyl acetate and lead acetate and examined under a transmission electron microscope (Hitachi H-600, Japan).

### 2.9. Isolation of Mitochondria and Cytosol

The mitochondrial and cytosolic fractions were prepared with the mitochondria/cytosol isolation kit (Applygen Technologies Inc., Beijing, China) according to the manufacturer's instructions [11]. All isolation procedures were conducted at 4°C. Heart tissues were homogenized with the ice-cold mitochondria/cytosol buffer as described above. After the first spin, the supernatant was further centrifuged at 12,000 g for 10 min to obtain pure cytosolic fraction. The pellet was resuspended in mitochondria/cytosol buffer and centrifuged at 12,000 g for 10 min again, and the final pellet was resuspended and represented the mitochondrial fraction. Both mitochondrial and cytosolic fractions were stored at −80°C until they were used. Protein content was determined by the BCA method as described above.

### 2.10. Cyt c Release Assay

The cytosolic fraction samples were normalized for protein and subjected to enzyme linked immunosorbent assay (ELISA) for estimating Cyt c contents following the manufacturer's instructions. Cyt c contents were analyzed at 450 nm and the optical density normalized to total protein content.

### 2.11. Determination of Mitochondrial Complex I Activity

The mitochondrial fraction samples were normalized for protein, and the activity of complex I was determined with a multimode microplate reader (Bio-Tek, Synergy HT, USA). Mitochondrial complex I was immunocaptured and the activity was determined at 450 nm by following the oxidation of nicotinamide adenine dinucleotide (NADH) to NAD^+^.

### 2.12. Measurement of Mitochondrial Swelling

The activation of the mPTP was determined by Ca^2+^-induced swelling of isolated cardiac mitochondria. The intact mitochondria samples were resuspended in swelling buffer containing (in mmol/L) 120 KCl, 5 KH_2_PO_4_, 20 MOPS, and 10 Tris*·*HCl, with pH 7.4 to a final protein concentration of 0.5 g/L. Mitochondrial permeability transition causes mitochondrial swelling, which is measured spectrophotometrically as a decrease in absorbance at 520 nm followed by the addition of 200 *μ*mol/L CaCl_2_ [12, 13].

### 2.13. Statistical Analysis

All data were expressed as mean ± SD and were subjected to one-way ANOVA followed by Bonferroni's multiple comparisons test. All results with *P* < 0.05 were considered statistically significant.

## 3. Results

### 3.1. SSNX Reduces Myocardial Infarct Size and Oxidative Stress and Preserves Left Ventricular Structure

**Figure 1:**
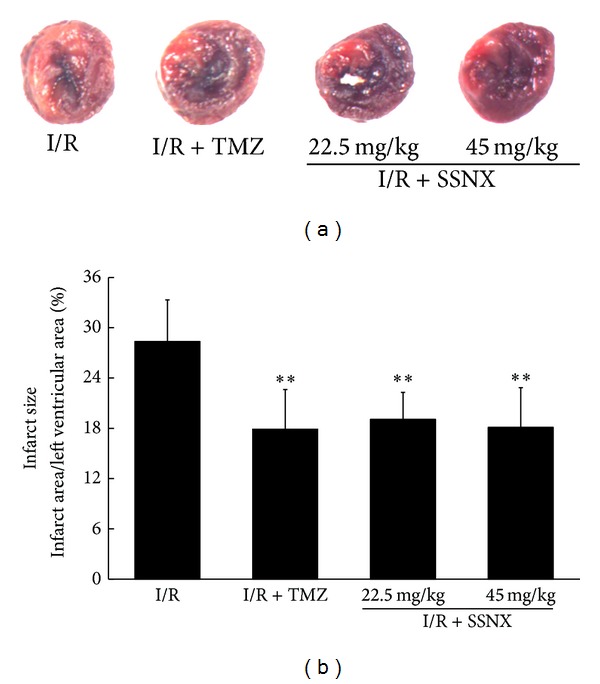
The effect of SSNX on infarct size after MI/R injury. (a) Representative midmyocardial cross-sections of NBT-stained hearts. The viable myocardium was stained dark blue, and the infracted myocardium was not stained. (b) Bar chart of myocardial infarct size determined by NBT staining. Data are shown as mean ± SD, *n* = 8/group. **P < 0.01 versus I/R group.

To examine whether SSNX treatment reduces the myocardial injury following I/R in rat hearts, myocardial infarct size was measured first. As shown in Figures 1(a) and 1(b), NBT staining of myocardium revealed that I/R challenged myocardium resulted in markedly increased percent infarct size (28.36 ± 4.96%). On the other hand, rats pretreatment with SSNX at dose of 22.5, 45 mg/kg, and TMZ at dose of 10 mg*·*kg^−1^ markedly reduced the infarct size as compared to I/R group (19.05 ± 3.24%, 18.10 ± 4.76%, and 17.89 ± 4.73%, respectively, versus I/R *P* < 0.01). The representative images of midventricular cross-sections from each group are shown in Figure 1(a).

Circulating plasma levels of LDH were evaluated as an additional marker of myocardial injury. As shown in Table 1, rats subjected to MI/R exhibited significant elevation of serum LDH activity as compared to the sham group (versus sham *P* < 0.01). However, as with the standard drug TMZ, pretreatment with SSNX dose dependently prevented the leakage of LDH from cardiomyocytes (versus I/R *P* < 0.05 or *P* < 0.01). Moreover, this preventative effect was associated with antioxidation. Table 1 also represents the effect of SSNX on the activity of antioxidant enzyme and lipid peroxidation marker in myocardial tissue. Rats in I/R group exhibited significant depletion in the activities of GSH-Px, with a concomitant increase in MDA levels as compared to the sham group (versus sham *P* < 0.01). However, pretreatment with SSNX for 5 days markedly reduced the production of MDA (versus I/R *P* < 0.01), and this preventive effect was partly associated with a significant increase in the GSH-Px activities (versus I/R *P* < 0.05), but pretreatment with SSNX at the dose of 22.5 mg/kg showed no significant effect on the GSH-Px activities.

**Figure 2:**
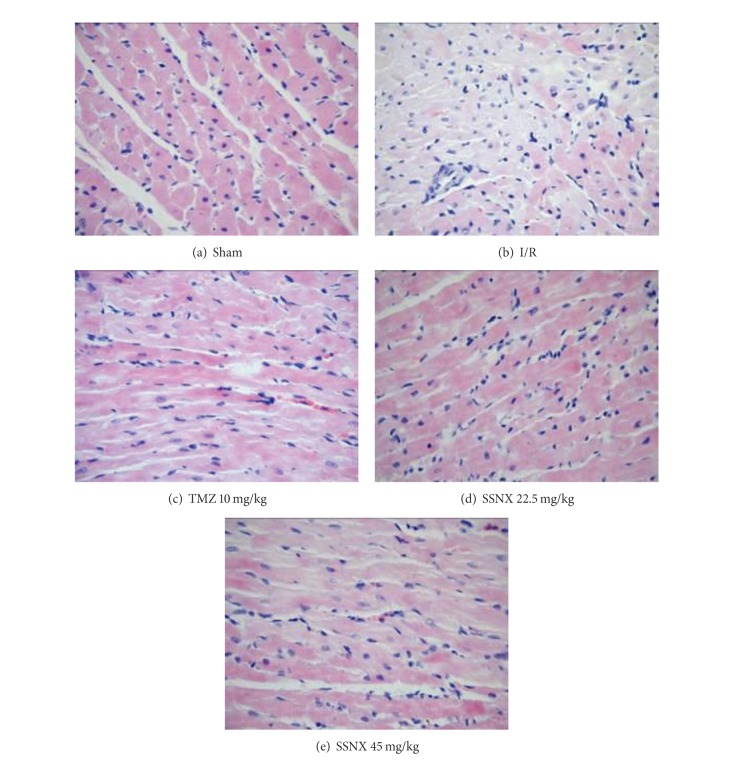
Representative HE-stained histological images after 40 min LCA ischemia and 2 h reperfusion (200x). *n* = 5/group.

Tissue sections were also stained with HE for the histopathological evaluation of hearts. As shown in Figure 2, I/R challenged hearts showed extensive myofibrillar degeneration, with infiltration of inflammatory cells and interstitial edema as compared with the sham group. TMZ and SSNX treated rats displayed a reduced degree of tissue edema, degenerative changes and disruption of myofibers, and inflammatory cell infiltration as compared to the sham group.

### 3.2. SSNX Reduces Cardiomyocyte Apoptosis after MI/R Injury

**Figure 3:**
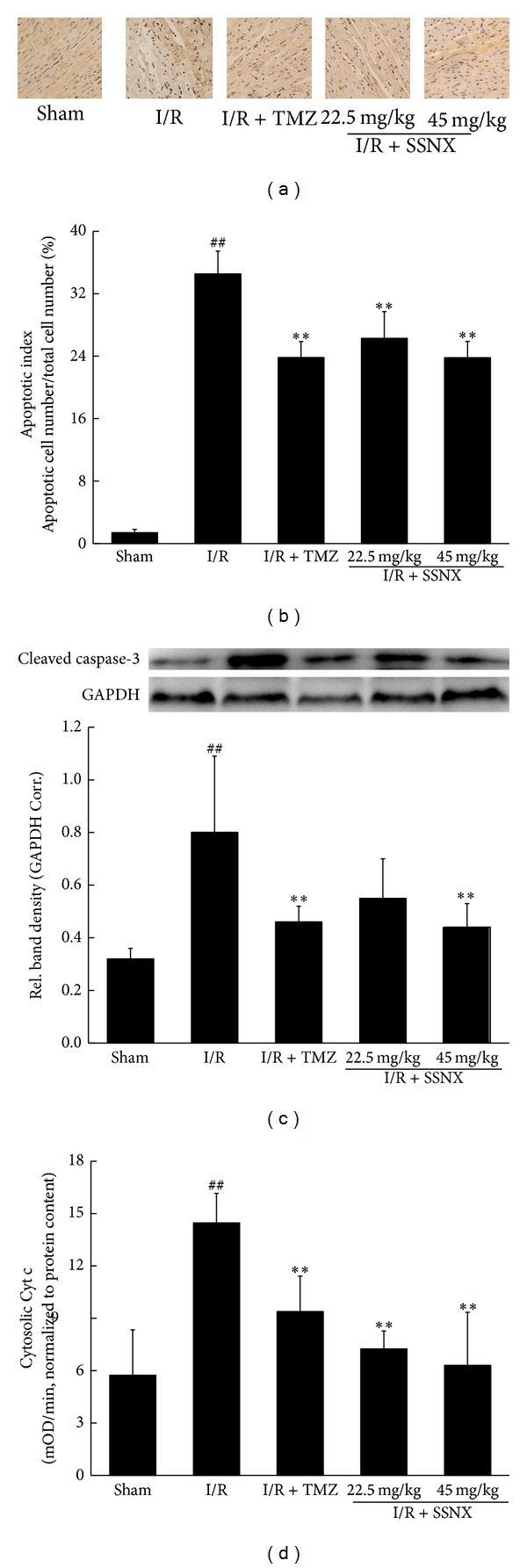
The effect of SSNX on cardiomyocyte apoptosis after MI/R injury. Representative photographs (a) and quantitative data (b) on cardiomyocyte apoptosis obtained by TUNEL staining, *n* = 5/group; (c) representative western blot images and densitometry analysis of immunoreactive bands of cleaved caspase-3, *n* = 6/group; (d) the effect of SSNX on cytosolic Cyt c levels after MI/R injury, *n* = 6/group. Data are shown as mean ± SD; ^##^ *P* < 0.01 versus sham group; **P < 0.01 versus I/R group.

Since inhibition of the apoptotic processes has been shown to prevent the MI/R injury, we hereby studied the effect of SSNX on apoptotic markers including TUNEL positivity and cleaved caspase-3 expressions' levels. As shown in Figures 3(a) and 3(b), the percentage of TUNEL-positive myocyte nuclei was markedly increased in I/R rats compared with the sham group (34.53 ± 2.92%, versus sham *P* < 0.01). As with the standard drug TMZ (23.81 ± 2.06%, versus I/R *P* < 0.01), rats' pretreatment with SSNX markedly reduced TUNEL-positive cells (26.28 ± 3.43% and 23.79 ± 2.10% versus I/R *P* < 0.01).

To further investigate the effects of SSNX on caspase-3 activation, we measured cleaved caspase-3 expression levels by western blot assay in tissue lysates. As shown in Figure 3(c), I/R caused a significant increase in cleaved caspase-3 expression as compared with that in the sham group (versus sham *P* < 0.01). On the other hand, rats treated with TMZ and SSNX at the dose of 45 mg/kg were found to have a significant decrease in cleaved caspase-3 expression levels (versus I/R *P* < 0.01).

Mitochondrial dysfunction may initiate apoptosis by releasing proapoptotic factors, such as Cyt c, from the mitochondrial intermembrane space into the cytosol to trigger apoptosis via a caspase-3-dependent pathway [14, 15]. Thus, changes in cytosolic Cyt c levels were measured by ELISA with the data normalized to total protein. As shown in Figure 3(d), the ELISA analysis revealed I/R group rats to have a significant increase in cytosolic Cyt c levels as compared with the sham group rats (versus sham *P* < 0.01) which indicated the release of Cyt c from the mitochondria into the cytosol, while this subcellular shift of Cyt c was markedly inhibited by pretreatment with SSNX and TMZ (versus I/R *P* < 0.01).

### 3.3. SSNX Preserves Mitochondrial Function and Membrane Integrity after MI/R Injury

**Figure 4:**
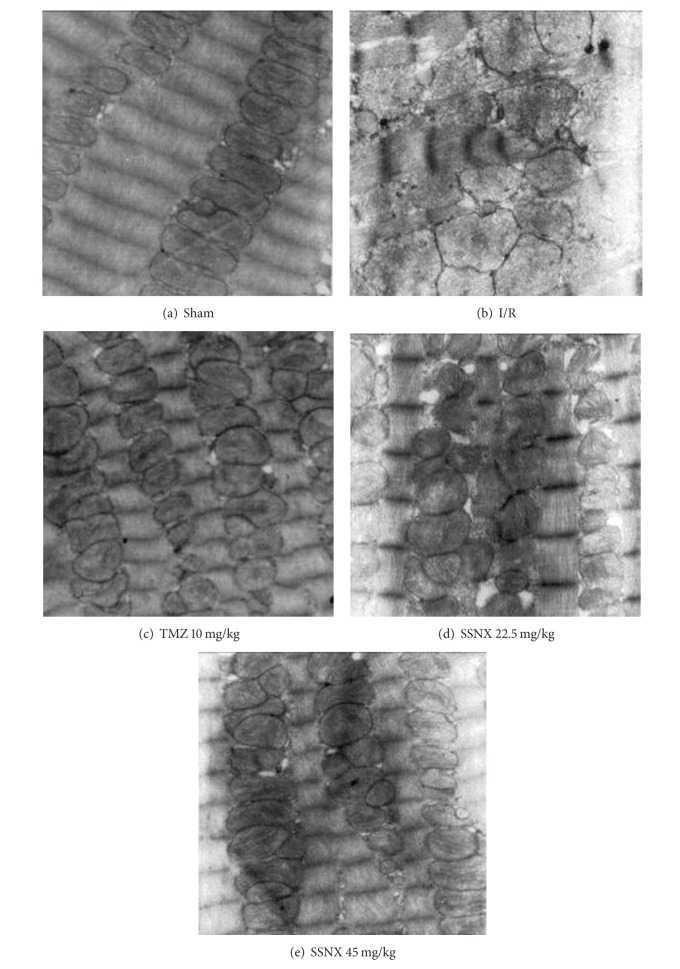
Representative electron micrographs of myocardial tissues after 40 min LCA ischemia and 2 h reperfusion (12,000x). *n* = 2/group.

After 40 min of ischemia and 2 h reperfusion, myocardial samples were also qualitatively assessed by transmission electron microscopy for structural mitochondrial changes. As shown in Figure 4, longitudinal sections of the I/R group hearts displayed uniform mitochondrial swelling with disorganized cristae and decreased matrix density, and the presence of amorphous matrix densities in a number of mitochondria revealed a distinctive feature of irreversible myocardial cell injury after reperfusion. TMZ and SSNX-pretreated hearts displayed little change in mitochondrial structure. Generally, mitochondria seemed to be highly dense with well-organized cristae and a decreased mitochondrial swelling as compared with I/R group myocardium.

**Figure 5:**
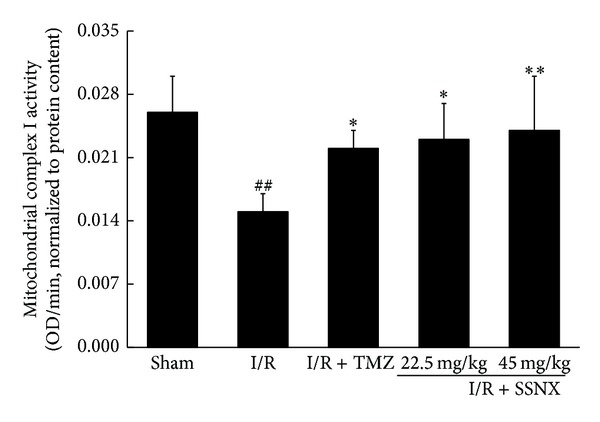
The effect of SSNX on mitochondrial complex I activity after MI/R injury. Data are shown as mean ± SD, *n* = 6/group. ^##^ *P* < 0.01 versus sham group; *P < 0.05 and **P < 0.01 versus I/R group.

The complex I (NADH-CoQ reductase) activities were measured in all the above preparations of mitochondria. As shown in Figure 5, mitochondria from I/R group rat hearts exhibited a marked decrease of 42% in the activities of complex I, compared with the sham group hearts (versus sham *P* < 0.01). As with the standard drug TMZ (versus I/R *P* < 0.05), rats pretreatment with SSNX at dose of 22.5, 45 mg/kg, had a protective effect and actually attenuated the decline in the complex I activities (versus I/R *P* < 0.05 and *P* < 0.01, resp.).

### 3.4. SSNX Reduces Mitochondrial Permeability Transition Induced by MI/R Injury

**Figure 6:**
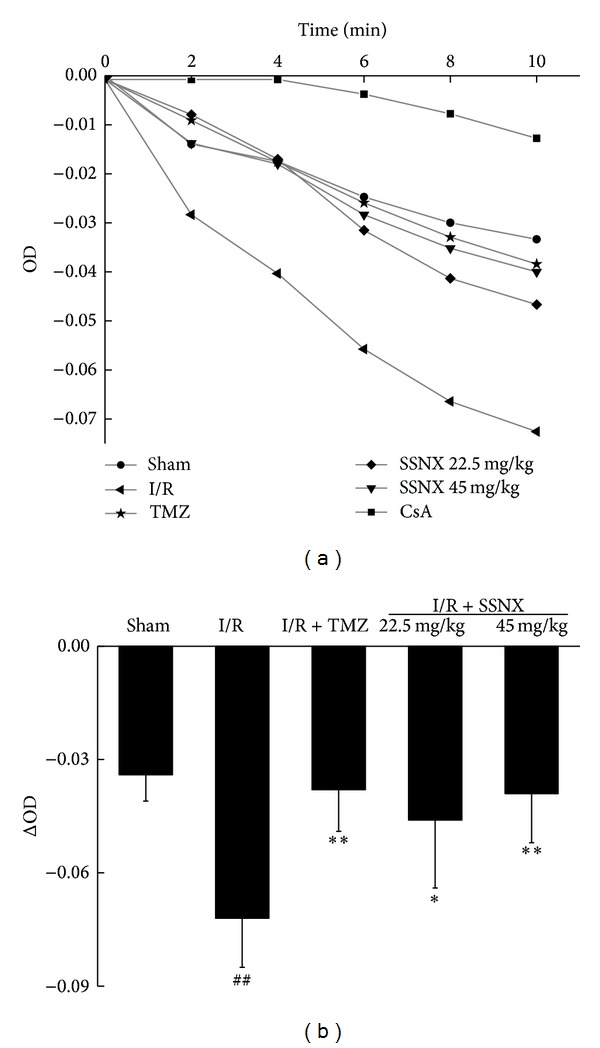
Effect of SSNX on mitochondrial permeability transition in cardiac tissue after MI/R injury. (a) Ca^2+^-induced mitochondrial swelling measured as decrease in the initial optical density (OD520) within 10 min. Note: cyclosporin A (CsA) is an mPTP inhibitor. (b) Mean changes in the initial optical density (OD520) at 10 min. Data are shown as mean ± SD, *n* = 6/group. ^##^ *P* < 0.01 versus sham group; *P < 0.05 and **P < 0.01 versus I/R group.

Mitochondrial swelling is a hallmark of mitochondrial dysfunction and is an important indicator of the opening of mPTP [16, 17]. To test if SSNX modulates the mitochondrial permeability transition, the mitochondrial swelling following calcium addition was measured as changes in absorbance in isolated mitochondria. As shown in Figure 6, MI/R induced an increase in the swelling rate of mitochondria by 215% compared with the sham group (versus sham *P* < 0.01). In contrast, pretreatment with TMZ and SSNX showed a much smaller increase in the swelling rate of mitochondria, implying that SSNX can prevent the mitochondrial permeability transition (versus I/R *P* < 0.05 and *P* < 0.01, resp.).

## 4. Discussion

First, TMZ was used as a positive control drug because it was a known metabolic anti-ischemic agent [18–20], which had a preferential action on mitochondrial function of ischemic hearts [21, 22]. In this study, we showed that pretreatment with SSNX for 5 days limited the extent of myocardial injury in a rat model in vivo. A dose-response study revealed that SSNX displayed a marked reduction in infarct size which had previously been reported by Liu et al. with a Chinese miniporcine myocardial ischemia model [7]. We also found that SSNX markedly reduced MI/R-induced cardiomyocyte apoptosis measured by TUNEL staining. Furthermore, the effects of SSNX against MI/R injury were confirmed by the improved histopathological changes and decreased intracellular LDH leakage. Importantly, the above protective effects were partly attributed to reduced oxidative stress in terms of decreasing MDA production and increasing GSH-Px activities.

Mitochondria play an important role in regulating the life and death of cells. They provide the cell with energy but can quickly switch from a supporter of life to a promoter of death in response to stress [23–25]. It is well established that myocardial ischemia and reperfusion are associated with mitochondrial dysfunction and cell death via both apoptosis and necrosis. Thus, another important research field in myocardial cytoprotection is the preservation of mitochondrial function [3, 4].

Complex I, also known as NADH-ubiquinone oxidoreductase, is a multisubunit integral membrane complex of the mitochondrial electron transport chain that catalyzes electron transfer from NADH to ubiquinone. Complex I is considered an important site of superoxide anion generation in mitochondria [26]. Moreover, it has been reported that MI/R injury resulted in a marked defect of complex I activities, of which the defect is the most important factor responsible for ROS production by increasing the electron leak from the electron transport chain [27]. This study demonstrated that mitochondria isolated from rats given TMZ displayed a significant increase in complex I activities, which is consistent with previously reported results [22]. Compared with TMZ, rats pretreated with SSNX were found to have a better recovery of complex I activities. This finding indicates that the reduced MDA production by treatment with SSNX may be partly ascribed to preservation of complex I activities, resulting in a decreased production of ROS.

In addition, a key aspect of mitochondrial involvement in cell demise is responsible for the opening of the mPTP [28]. The mPTP is a highly dynamic, nonselective pore which is thought to mediate the lethal permeability changes of the outer mitochondrial membrane (OMM) and the inner mitochondrial membrane (IMM) that causes release of proapoptotic proteins and loss of membrane potential that may lead to apoptotic cell death [24, 29]. It is proposed that MI/R promotes mPTP opening, especially in reperfusion conditions such as increased matrix Ca^2+^, Pi, and ROS which favor long-lasting pore opening, resulting in swelling and subsequent rupture of mitochondria [30]. In this study, electron microscopy revealed a striking reduction in mitochondrial swelling and increased matrix density in rats receiving SSNX, suggesting a prominent role for the preservation of mitochondrial function in the observed cytoprotection. Furthermore, mPTP displayed an increased sensitivity to calcium after MI/R, and pretreatment with SSNX largely delayed MI/R-induced mitochondrial permeability transition. As has been established, the permeabilization of the OMM results in the release of pro-death proteins, such as Cyt c, from the mitochondrial intermembrane space into the cytosol [28, 31]. Once it is released into the cytosol, Cyt c triggers the formation of Apaf-1/caspase-9 apoptosome and activation of caspases to initiate apoptosis [31], and caspase-3 has conventionally been considered one of the executioner caspases. A further exploration of the role of SSNX involved in apoptotic processes reveals that SSNX inhibits the activation of caspase-3, which is associated with lower cytosolic Cyt c levels. Although the precise mechanisms require further study, this study demonstrates that SSNX exerts an antiapoptotic effect by blocking the mitochondrial apoptotic pathway.

In conclusion, we have shown that pretreatment with SSNX limits the extent of myocardial injury. This protection is accompanied by a decrease in myocardial oxidative stress and cardiomyocyte apoptosis, and these are associated with preservation of mitochondrial function. Furthermore, the present results can also partly account for the improved cardiac energy metabolism of the SSNX treated rat heart after MI/R injury [10], which is reported in our previous studies. Thus, preservation of mitochondrial function may be a new mechanism by which SSNX exerts its cardioprotective effects. In addition, another important mechanism of cardiomyocyte protection by SSNX that we have found in previous studies is suppression of hypoxia/reoxygenation induced-calcium overload [32–34]. These studies confirm that the antimyocardial ischemia effect of SSNX is achieved through collectively modulating the multitargets of the body system by its active ingredients, which is one of the significant advantages of TCM in treating complex diseases.

## Figures and Tables

**Table 1:** Theeffect of SSNX on serum LDH levels, MDA contents and GSH-Px activities after MI/R injury.

Groups	Dose, mg/kg	LDH, U/L	MDA, nmol/mg protein	GSH-Px, U/mg protein
Sham	—	489 ± 96	1.58 ± 0.25	29.93 ± 6.11
I/R	—	1843 ± 787^##^	4.55 ± 0.81^##^	16.14 ± 2.32^##^
I/R + TMZ	10	911 ± 416∗∗	1.89 ± 0.26∗∗	24.77 ± 3.91∗
I/R + SSNX	22.5	953 ± 271∗	2.32 ± 0.89∗∗	22.14 ± 4.47
I/R + SSNX	45	828 ± 301∗∗	2.17 ± 0.49∗∗	25.10 ± 5.52∗

Note: LDH, *n* = 8/group; MDA, *n* = 6/group; GSH-Px, *n* = 6/group; Date are shown as mean ± SD, ^##^ *P* < 0.01 versus Sham group, ∗*P* < 0.05, ∗∗*P* < 0.01 versus I/R group.
